# Zonotope-Based State Estimation for Boost Converter System with Markov Jump Process

**DOI:** 10.3390/mi16101099

**Published:** 2025-09-27

**Authors:** Chaoxu Guan, You Li, Zhenyu Wang, Weizhong Chen

**Affiliations:** 1 College of Mechanical Engineering, Jiaxing University, Jiaxing 314001, China; guanchaoxu@sina.com; 2 Zhejiang Academy of Special Equipment Science, Hangzhou 310020, China; 3 School of Electronics and Information, Xi’an Polytechnic University, Xi’an 710048, China; chenweizhong124@163.com; 4 Xi’an Polytechnic University Branch of Shaanxi Artificial Intelligence Joint Laboratory, Xi’an 710048, China

**Keywords:** Markov jump process, boost converter, state estimation, adaptive event-triggered mechanism, zonotopes

## Abstract

This article investigates the zonotope-based state estimation for boost converter system with Markov jump process. DC-DC boost converters are pivotal in modern power electronics, enabling renewable energy integration, electric vehicle charging, and microgrid operations by elevating low input voltages from sources like photovoltaics to stable high outputs. However, their nonlinear dynamics and sensitivity to uncertainties/disturbances degrade control precision, driving research into robust state estimation. To address these challenges, the boost converter is modeled as a Markov jump system to characterize stochastic switching, with time delays, disturbances, and noises integrated for a generalized discrete-time model. An adaptive event-triggered mechanism is adopted to administrate the data transmission to conserve communication resources. A zonotopic set-membership estimation design is proposed, which involves designing an observer for the augmented system to ensure $H_{\infty }$ performance and developing an algorithm to construct zonotopes that enclose all system states. Finally, numerical simulations are performed to verify the effectiveness of the proposed approach.

## 1. Introduction

DC-DC boost converters have emerged as pivotal components in modern power electronic systems, particularly in renewable energy integration, electric vehicle charging, and microgrid applications [1,2,3,4,5]. These converters play a critical role in stepping up low input voltages from sources such as photovoltaic panels, fuel cells, and batteries to a stable, higher output voltage required by downstream devices. However, their inherent nonlinear dynamics—characterized by non-minimum phase behavior, bilinear characteristics, and time-varying parameters (e.g., input voltage fluctuations and load variations)—pose significant challenges to precise control and regulation. The performance of boost converters is highly sensitive to parametric uncertainties and external disturbances, which can degrade voltage stability, increase transient errors, and even lead to system instability if not properly addressed. In recent years, a number of research efforts have been made to tackle these challenges specific to boost converters. For example, a model-free control scheme is developed for single-phase boost converters operating under disturbances and uncertainties in [6]. By utilizing a graphical strategy, a controller tuning approach is proposed for a DC-DC boost converter with the system’s stability and robustness being addressed through the acquisition of the maximum sensitivity region in [7]. In [8], the authors incorporate mismatching disturbance compensation into robust $H_{\infty }$ controller to improve a boost converter’s robustness and disturbance rejection capability. Nevertheless, to date, the body of relevant findings remains relatively limited. Developing robust strategies to handle these complexities is essential for ensuring reliable operation in diverse engineering scenarios.

State estimation and filtering techniques have become indispensable tools for enhancing the performance and robustness of various dynamic systems [9,10,11,12]. In practical applications, direct measurement of key states (e.g., inductor current, capacitor voltage) and parameters (e.g., load resistance, input voltage) is often hindered by sensor noise, cost constraints, or physical inaccessibility. State estimation methods enable real-time reconstruction of unmeasured states or uncertain parameters using available measurements, which not only mitigate the impact of noise and disturbances but also provide critical information for feedback control, allowing the system to adapt to varying operating conditions. For instance, estimation-based control strategies can compensate for load changes, input voltage fluctuations, and component tolerances, thereby improving transient response and steady-state accuracy. Among state estimation techniques, the set-membership approach has garnered significant attention in recent years, primarily due to its capacity to generate compact sets, such as intervals, ellipsoids, and zonotopes, that contain all permissible values of the system’s state [13,14,15]. Typically, the interval observer method obtains the upper and lower bounds of system state estimates by designing two distinct sub-observers, which imposes a key requirement that the error system must be cooperative. This condition is highly restrictive and often challenging to meet in practical applications. In contrast to the interval observer method, the zonotopic set-membership approach offers more accurate estimation outcomes [16]. Furthermore, the zonotope-based method features a relatively straightforward iteration structure, which enables it to suppress wrapping effects and reduce computational burdens. Owing to these advantages, the zonotopic estimation method has become a preferred choice in theoretical research and engineering practice [17,18,19]. In [20], the zonotope-based non-fragile fusion estimation issue is addressed for nonlinear systems subject to sensor resolution impacts. By virtue of zonotopic reachability analysis, a resilient state estimation is investigated for unmanned surface vehicles and a segment minimization approach is presented for the optimization of the system state set [21]. Leveraging the zonotopic estimation technique, the authors in [22] establish a fault diagnosis scheme for the fundamental microbial growth process in wastewater treatment.

On the other hand, sudden environmental shifts, system perturbations, and subsystem malfunctions frequently introduce complexities into numerous practical engineering systems, posing notable challenges to their analysis and control. In this context, Markov jump processes (MJPs) emerge as an important tool for modeling such systems. MJPs have proven highly effective in addressing practical problems due to their ability to capture abrupt phenomena like random component failures and repairs, sudden environmental alterations, the dynamics in networked control systems, etc. [23,24,25]. In [26], a robust recursive regulation issue is studied for dynamic systems with MJPs, which is further applied to autonomous vehicle control synthesis. A saturated-threshold event-triggered control strategy is proposed for nonlinear systems with MJPs, validating their usefulness and practicality through a chemical reactor case [27]. By employing T-S fuzzy model, an observer-based sliding mode control is addressed for stochastic nonlinear Markov jump systems with application to single-link robot arms [28]. When it comes to power electronics, specifically in modeling the switching behavior of boost converters, MJPs hold great promise. The switching in boost converters is subject to various random factors, and MJPs can accurately characterize these stochastic switching behaviors, thereby facilitating the analysis and synthesis of boost converter systems [29,30,31].

The present paper is devoted to the state estimation for boost converter systems by a zonotope-based method. Firstly, aiming at the stochastic switching behaviors and the impact of time delays, disturbances, and noises in boost converters, we establish a generalized discrete-time state space model by formulating the boost converter as a Markov jump system. Secondly, considering the demand for efficient communication resource utilization in practical applications, an adaptive event-triggered mechanism is introduced to manage data sending. Then, concentrating on robust state estimation, we put forward a zonotopic set-membership estimation design. This design encompasses two main aspects: one is devising an observer to achieve $H_{\infty }$ performance, which is crucial for suppressing the influence of disturbances and noises; the other is developing a dedicated algorithm to construct zonotopes that can contain all possible system states, thus providing a reliable bounding estimation. Finally, the validity of the proposed approach is demonstrated through numerical simulations. The organization of this paper is as follows. Section 2 introduces the boost converter model with its mathematical transformation and gives some preliminaries. In Section 3, the main results of this work are proposed, including $H_{\infty }$ observer design and zonotopic estimation strategy. Then Section 4 illustrates the effectiveness of the proposed method via simulation experiments. Section 5 concludes this paper and discusses potential future research. The notations throughout this work are expressed in Table 1.

## 2. Model Descriptions and Preliminaries

### 2.1. Boost Converter System

The boost converter system is a kind of power electronic converter that can increase the output voltage level. It finds extensive applications in various power supply setups such as single-phase power correction circuits and DC motor drives. By regulating the switching action, this system enables the output voltage to be higher than the input voltage.

**Figure 1:**
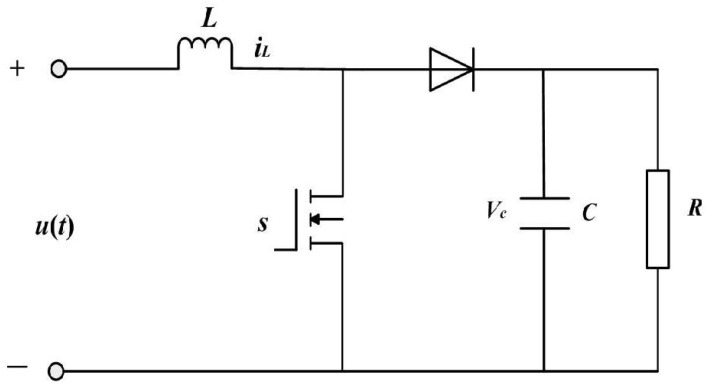
Circuit diagram of the boost converter.

Figure 1 displays the circuit principle of a boost converter system with reference to [30]. In this model, $V_{c}$ represents the voltage across capacitance *C*, $i_{L}$ denotes the current flowing through inductance *L*, symbol *s* is the transfer switch, and u(t) is the control input signal. Different connection states of the transfer switch result in different modes of the system. If the transfer switch is open, the system is in the first mode and its dynamic equation is described by

(1) $$\begin{array}{c} \left\{\begin{array}{c} C\frac{dV_{c}}{dt}=i_{L}-\frac{V_{c}}{R},\\ L\frac{di_{L}}{dt}=u\left(t\right)-V_{c}. \end{array}\right. \end{array}$$

If the transfer switch is closed, the system is in the second mode and its dynamic equation is described by

(2) $$\begin{array}{c} \left\{\begin{array}{c} RC\frac{dV_{c}}{dt}=-V_{c},\\ L\frac{di_{L}}{dt}=u\left(t\right). \end{array}\right. \end{array}$$

Considering the complexity of the real-world environment, the system parameters or structure may undergo abrupt and random changes. Here, we employ a Markov jump process σ(t) to model the switching among different modes in the boost converter system. By defining $x\left(t\right)={\left[V_{c},i_{L}\right]}^{T}$ as the state variables, the nominal state space model can be formulated by

(3) $$\begin{array}{c} \left\{\begin{array}{c} \dot{x}\left(t\right)=A_{\sigma (t)}x\left(t\right)+B_{\sigma (t)}u\left(t\right),\\ y\left(t\right)=C_{\sigma (t)}x\left(t\right), \end{array}\right. \end{array}$$

where y(t) is the measurement output and

$$\begin{array}{cc} A_{1}= & \left[\begin{array}{cc} -\frac{1}{RC} & \frac{1}{C}\\ -\frac{1}{L} & 0 \end{array}\right],A_{2}=\left[\begin{array}{cc} -\frac{1}{RC} & 0\\ 0 & 0 \end{array}\right],B_{\sigma (t)}=\left[\begin{array}{c} 0\\ \frac{1}{L} \end{array}\right],\\ C_{\sigma (t)}= & \left[\begin{array}{cc} 1 & 0 \end{array}\right],\sigma \left(t\right)\in £=\left\{1,2\right\}. \end{array}$$

### 2.2. Generalized Mathematical Description

Given that time delays and system uncertainties including disturbances and noises are prevalent in practical scenarios, this study incorporates these factors to obtain a more generalized research model. By the discretization technique, the generalized mathematical model for boost converter systems is described by the following formula with Markov jump process:

(4) $$\begin{array}{c} \left\{\begin{array}{c} x_{k+1}=\sum_{t=0}^{h}\left(A_{t,\sigma (k)}x_{k-t}\right)+B_{\sigma (k)}u_{k}+E_{\sigma (k)}w_{k},\\ y_{k}=C_{\sigma (k)}x_{k}+F_{\sigma (k)}v_{k}, \end{array}\right. \end{array}$$

where $x_{k}\in R^{n_{x}},u_{k}\in R^{n_{u}},y_{k}\in R^{n_{y}}$ are the state, input, and measurement output vectors, respectively. $w_{k}\in R^{n_{w}}$ denotes the external disturbance and $v_{k}\in R^{n_{v}}$ means the measurement noise. The discrete-time Markov chain is represented by σ(k) taking values in a finite set £={1,2,⋯,N}. $A_{t,\sigma (k)},B_{\sigma (k)},E_{\sigma (k)},C_{\sigma (k)},F_{\sigma (k)}$ are known constant matrices with proper dimensions and abbreviated as $A_{t,i},B_{i},E_{i},C_{i},F_{i}$ for σ(k)=i∈£. The transition probability matrix is denoted by $\Gamma ={\left[\mu_{ij}\right]}_{N\times N}$ with

$$\begin{array}{c} Pr\left\{\sigma \left(k+1\right)=j|\sigma \left(k\right)=i\right\}=\mu_{ij},\forall i,j\in £, \end{array}$$

and

$$\begin{array}{c} 0\leq \mu_{ij}\leq 1,\underset{j=1}{\sum^{N}}\mu_{ij}=1. \end{array}$$

The coupling introduced by multiple time delays increases difficulties in system analysis. To handle the undesired impact, the state augmentation method is employed here to transform system (4) into an augmented version without time delays.

According to system (4), we have

(5) $$\begin{array}{c} \left\{\begin{array}{c} x_{k+1}=\sum_{t=0}^{h}\left(A_{t,i}x_{k-t}\right)+B_{i}u_{k}+E_{i}w_{k},\\ x_{k}=x_{k},\\ \vdots \\ x_{k-h}=x_{k-h},\\ y_{k}=C_{i}x_{k}+F_{i}v_{k}. \end{array}\right. \end{array}$$

Denote the augmented state vector ${\bar{x}}_{k}$ as

$$\begin{array}{c} {\bar{x}}_{k}={\left[x_{k}^{T},x_{k-1}^{T},\cdots ,x_{k-h}^{T}\right]}^{T}\in R^{\left(h+1\right)n_{x}}. \end{array}$$

Then the system (5) is reformulated as the following form without time delays:

(6) $$\begin{array}{c} \left\{\begin{array}{c} {\bar{x}}_{k+1}={\bar{A}}_{i}{\bar{x}}_{k}+{\bar{B}}_{i}u_{k}+{\bar{E}}_{i}w_{k},\\ y_{k}={\bar{C}}_{i}{\bar{x}}_{k}+F_{i}v_{k}, \end{array}\right. \end{array}$$

where

$$\begin{array}{cc} {\bar{A}}_{i}= & \left[\begin{array}{cccc} A_{0,i} & \cdots & A_{h-1,i} & A_{h,i}\\ I_{n_{x}} & \cdots & 0 & 0\\ \vdots & \; & \vdots & \vdots \\ 0 & \cdots & I_{n_{x}} & 0 \end{array}\right],{\bar{B}}_{i}=\left[\begin{array}{c} B_{i}\\ 0\\ \vdots \\ 0 \end{array}\right],\\ {\bar{E}}_{i}= & \left[\begin{array}{c} E_{i}\\ 0\\ \vdots \\ 0 \end{array}\right],{\bar{C}}_{i}={\left[\begin{array}{c} C_{i}^{T}\\ 0\\ \vdots \\ 0 \end{array}\right]}^{T}. \end{array}$$

### 2.3. Adaptive Event-Triggered Observer

**Figure 2:**
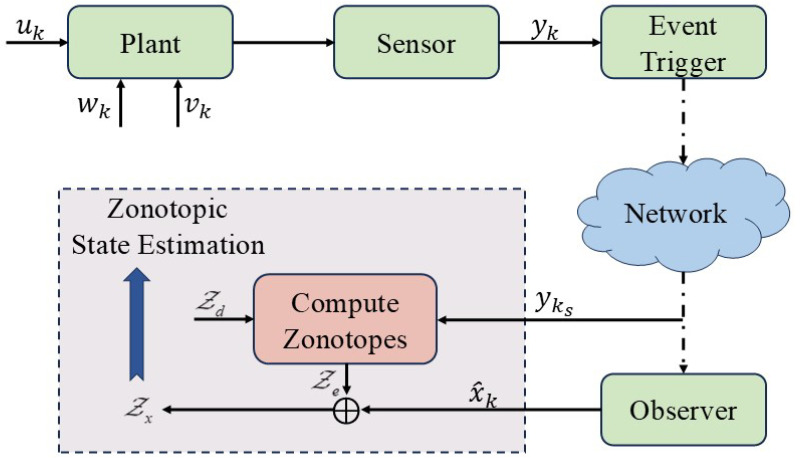
Adaptive event-triggered mechanism for zonotopic state estimation.

Bearing in mind the burden of communication, an adaptive event-triggered mechanism is considered to administrate the signal transmission as shown in Figure 2. Define the triggering instants as $\left\{k_{0},\cdots ,k_{s},k_{s+1},\cdots \right\}$ with $k_{0}=0$. The adaptive event-triggered mechanism is formulated by

(7) $$\begin{array}{c} k_{s+1}=\underset{k}{\inf}\left\{k>k_{s}|\frac{1}{\theta }q_{k}+\varepsilon y_{k}^{T}y_{k}<\delta_{k}^{T}\delta_{k}\right\}, \end{array}$$

where $\delta_{k}=y_{k}-y_{k_{s}}$ and $q_{k}$ serves as the adaptive parameter whose dynamics follow the rule as below:

(8) $$\begin{array}{c} q_{k+1}=\tau q_{k}+\varepsilon y_{k}^{T}y_{k}-\delta_{k}^{T}\delta_{k},k\in \left[k_{s},k_{s+1}\right), \end{array}$$

with 0<τ,ε<1,θ>0 and the initial value $q_{0}\geq 0$. Accounting for the behavior of ZOH, the input signal of the event-triggered observer is expressed as

$$\begin{array}{c} {\bar{y}}_{k}=y_{k_{s}},k\in \left[k_{s},k_{s+1}\right). \end{array}$$

Then the event-triggered observer is established as

(9) $$\begin{array}{c} {\hat{x}}_{k+1}={\bar{A}}_{i}{\hat{x}}_{k}+{\bar{B}}_{i}u_{k}+L_{i}\left({\bar{y}}_{k}-{\bar{C}}_{i}{\hat{x}}_{k}\right), \end{array}$$

where ${\hat{x}}_{k}$ is the observer state and $L_{i}$ is the observer gain to be designed. By defining the estimation error $e_{k}={\bar{x}}_{k}-{\hat{x}}_{k}$, the dynamics of the error system can be described as

(10) $$\begin{array}{c} e_{k+1}=A_{e,i}e_{k}+D_{i}d_{k}+L_{i}\delta_{k}, \end{array}$$

where

$$\begin{array}{c} A_{e,i}={\bar{A}}_{i}-L_{i}{\bar{C}}_{i},D_{i}=\left[\begin{array}{cc} {\bar{E}}_{i} & -L_{i}F_{i} \end{array}\right],d_{k}=\left[\begin{array}{c} w_{k}\\ v_{k} \end{array}\right]. \end{array}$$

Denote $\xi_{k}=\left[\begin{array}{c} e_{k}\\ {\bar{x}}_{k} \end{array}\right],\eta_{k}=\left[\begin{array}{c} d_{k}\\ u_{k} \end{array}\right]$. Combining (6) and (10) implies the augmented Markov jump system as below

(11) $$\begin{array}{c} \left\{\begin{array}{c} \xi_{k+1}=A_{i}\xi_{k}+B_{i}\eta_{k}+E_{i}\delta_{k},\\ y_{k}=C_{i}\xi_{k}+D_{i}\eta_{k}, \end{array}\right. \end{array}$$

where

$$\begin{array}{cc} A_{i}= & \left[\begin{array}{cc} A_{e,i} & 0\\ 0 & {\bar{A}}_{i} \end{array}\right],B_{i}=\left[\begin{array}{ccc} {\bar{E}}_{i} & -L_{i}F_{i} & 0\\ {\bar{E}}_{i} & 0 & {\bar{B}}_{i} \end{array}\right],E_{i}=\left[\begin{array}{c} L_{i}\\ 0 \end{array}\right],\\ C_{i}= & \left[\begin{array}{cc} 0 & {\bar{C}}_{i} \end{array}\right],D_{i}=\left[\begin{array}{ccc} 0 & F_{i} & 0 \end{array}\right]. \end{array}$$

**Remark** **1.** *It is worth noting that the conventional event-triggered mechanism is termed static because it merely takes into account the values of $y_{k}$ and $\delta_{k}$. In this article, we adopt the adaptive event-triggered mechanism (7), in which an extra dynamic variable $q_{k}$ that conforms to a specific adaptive rule is incorporated into the triggering condition. Moreover, according to the condition (7), it can be obtained that for $\forall k\in [k_{s},k_{s+1})$,*

$$\begin{array}{c} \frac{1}{\theta }q_{k}+\varepsilon y_{k}^{T}y_{k}\geq \delta_{k}^{T}\delta_{k}, \end{array}$$

*which thus yields*

$$\begin{array}{c} q_{k+1}\geq \left(\tau -\frac{1}{\theta }\right)q_{k}. \end{array}$$

*By selecting parameters appropriately such that $\tau -\frac{1}{\theta }>0$ and $q_{0}\geq 0$, it is easy to get*

$$\begin{array}{c} q_{k}\geq {\left(\tau -\frac{1}{\theta }\right)}^{k}q_{0}\geq 0. \end{array}$$

**Lemma** **1.** *For the real-valued matrices U,V, and P having compatible dimensions, where P is a symmetric and positive-definite matrix, the subsequent inequality is satisfied*

$$\begin{array}{c} V^{T}U+U^{T}V\leq V^{T}P+U^{T}P^{-1}U. \end{array}$$

**Definition** **1.** *A zonotope Z=〈p,H〉, having a center $p\in R^{m}$ and a generator matrix $H\in R^{m\times \kappa }$, is defined as an affine transformation of a hypercube $B^{\kappa }={\left[-1,+1\right]}^{\kappa }$:*

(12) $$\begin{array}{c} Z=p⊕HB^{\kappa }=\left\{p+Hz:z\in B^{\kappa }\right\}. \end{array}$$

**Lemma** **2.** *Given some centers and generator matrices of zonotopes as $p,p_{1},p_{2}\in R^{m}$ and $H,H_{1},H_{2}\in R^{m\times \kappa }$, $T\in R^{t\times m}$ is a matrix with proper dimensions. The following characteristics of zonotopes are satisfied:*

$$\begin{array}{cc} \; & \langle p_{1},H_{1}\rangle ⊕\langle p_{2},H_{2}\rangle =\langle p_{1}+p_{2},\left[H_{1},H_{2}\right]\rangle ,\\ \; & T⊙\langle p,H\rangle =\langle Tp,TH\rangle ,\\ \; & \langle p,H\rangle \in \langle p,rs(H)\rangle , \end{array}$$

*where $rs\left(H\right)=diag\left\{h_{1},\cdots ,h_{m}\right\}$ and $h_{i}=\sum_{k=1}^{\kappa }\left|H_{i,k}\right|,i=1,\cdots ,m$.*

## 3. Zonotope-Based State Estimation

This paper presents a two-step design for the zonotope-based estimation for Markov jump systems, including the event-triggered observer design with $H_{\infty }$ performance and the zonotopic set-valued estimation within the algorithm. More precisely, our purposes can be summarized as follows:

**(i)** Design an observer for system (11) to ensure that vector $\xi_{k}$ has an $H_{\infty }$ performance in defense of uncertainties. In other words, for a constant γ>0 and nonzero $\eta_{k}\in L_{2}\left[0,\infty \right)$, the following inequality holds under zero initial conditions:

(13) $$\begin{array}{c} E\{\underset{k=0}{\sum^{\infty }}∥\xi_{k}{∥}^{2}\}\leq \gamma^{2}\underset{k=0}{\sum^{\infty }}{∥\eta_{k}∥}^{2}. \end{array}$$

**(ii)** Devise an algorithm for constructing a state-bounding zonotope that encloses all the system states within specified compact sets.

### 3.1. $H_{\infty }$ Observer Design

**Theorem** **1.** *Given a scalar γ>0, the augmented Markov jump system in (11) is stochastically stable with an $H_{\infty }$ performance γ if there exist matrices $P_{i}>0,i\in £$ such that for any i∈£,*

(14) $$\begin{array}{c} \Pi_{i}=\left[\begin{array}{ccc} \Pi_{i}^{11} & \Pi_{i}^{12} & \Pi_{i}^{13}\\ {}* & \Pi_{i}^{22} & \Pi_{i}^{23}\\ {}* & * & \Pi_{i}^{33} \end{array}\right]<0 \end{array}$$

*where*

$$\begin{array}{cc} \Pi_{i}^{11}= & A_{i}^{T}\left(\sum_{j\in £}\mu_{ij}P_{j}\right)A_{i}-P_{i}+\bar{q}\varepsilon C_{i}^{T}C_{i}+I,\\ \Pi_{i}^{12}= & A_{i}^{T}\left(\sum_{j\in £}\mu_{ij}P_{j}\right)B_{i}+\bar{q}\varepsilon C_{i}^{T}D_{i},\\ \Pi_{i}^{22}= & B_{i}^{T}\left(\sum_{j\in £}\mu_{ij}P_{j}\right)B_{i}+\bar{q}\varepsilon D_{i}^{T}D_{i}-\gamma^{2}I,\\ \Pi_{i}^{13}= & A_{i}^{T}\left(\sum_{j\in £}\mu_{ij}P_{j}\right)E_{i},\\ \Pi_{i}^{23}= & B_{i}^{T}\left(\sum_{j\in £}\mu_{ij}P_{j}\right)E_{i},\\ \Pi_{i}^{33}= & E_{i}^{T}\left(\sum_{j\in £}\mu_{ij}P_{j}\right)E_{i}-\bar{q}I,\\ \bar{q}= & \frac{1}{\theta }+1-\tau . \end{array}$$

**Proof.**  For system (11), construct the Lyapunov function candidate as $V\left(k\right)=V_{1}\left(k\right)+V_{2}\left(k\right)$ with

(15) $$\begin{array}{cc} V_{1}\left(k\right)= & \xi_{k}^{T}P_{i}\xi_{k}, \end{array}$$

(16) $$\begin{array}{cc} V_{2}\left(k\right)= & \frac{1}{\theta }q_{k}. \end{array}$$

Define

$$\begin{array}{c} \Delta V\left(k\right)=E\left\{V\left(\xi_{k+1},\sigma \left(k+1\right)|\xi_{k},\sigma \left(k\right)\right)\right\}-V\left(\xi_{k},\sigma \left(k\right)\right). \end{array}$$

Then we have

$$\begin{array}{c} \Delta V_{1}\left(k\right)=\xi_{k+1}^{T}\left(\sum_{j\in £}\mu_{ij}P_{j}\right)\xi_{k+1}-\xi_{k}^{T}P_{i}\xi_{k}. \end{array}$$

Combining the property of the adaptive parameter $q_{k}$, it follows that

$$\begin{array}{cc} \Delta V_{2}\left(k\right)= & \frac{1}{\theta }\left(\tau q_{k}+\varepsilon y_{k}^{T}y_{k}-\delta_{k}^{T}\delta_{k}-q_{k}\right)\\ \leq & \left(\tau -\frac{1}{\theta }-1\right)\delta_{k}^{T}\delta_{k}-\left(\tau -\frac{1}{\theta }-1\right)\varepsilon y_{k}^{T}y_{k} \end{array}$$

For zero initial values, we define

$$\begin{array}{c} J=E\{\underset{k=0}{\sum^{\infty }}\xi_{k}^{T}\xi_{k}-\gamma^{2}\eta_{k}^{T}\eta_{k}\}. \end{array}$$

The following inequality holds:

$$\begin{array}{cc} J\leq & \underset{k=0}{\sum^{\infty }}\left[\xi_{k}^{T}\xi_{k}-\gamma^{2}\eta_{k}^{T}\eta_{k}+\Delta V\left(k\right)\right]\\ = & \underset{k=0}{\sum^{\infty }}\chi_{k}^{T}\Pi_{i}\chi_{k}, \end{array}$$

where $\chi_{k}={\left[\begin{array}{ccc} \xi_{k}^{T} & \eta_{k}^{T} & \delta_{k}^{T} \end{array}\right]}^{T}$. It follows readily from condition (14) that J<0. Accordingly, the stochastic stability with an $H_{\infty }$ performance γ is derived for the system (11), which thus finishes the proof.    □

Based on the $H_{\infty }$ performance analysis in Theorem 1, the subsequent task is to design the observer gain for system (11) to ensure the optimal $H_{\infty }$ performance.

**Theorem** **2.** *Given a scalar γ>0, the augmented Markov jump system in (11) is stochastically stable with an $H_{\infty }$ performance γ if there exist matrices $P_{i}>0$, invertible matrices $U_{i}=\left[\begin{array}{cc} U_{i}^{1} & 0\\ 0 & U_{i}^{2} \end{array}\right]$, and $K_{i},i\in £$ such that for any i∈£,*

(17) $$\begin{array}{c} \left[\begin{array}{cccccc} \Psi_{i1} & 0 & \cdots & \sqrt{\mu_{i1}}\Phi_{i}^{1} & \sqrt{\mu_{i1}}\Phi_{i}^{2} & \sqrt{\mu_{i1}}\Phi_{i}^{3}\\ {}* & \vdots & \vdots & \vdots & \vdots & \vdots \\ {}* & * & \Psi_{iN} & \sqrt{\mu_{iN}}\Phi_{i}^{1} & \sqrt{\mu_{iN}}\Phi_{i}^{2} & \sqrt{\mu_{iN}}\Phi_{i}^{3}\\ {}* & * & * & \Xi_{i}^{1} & \bar{q}\varepsilon C_{i}^{T}D_{i} & 0\\ {}* & * & * & * & \Xi_{i}^{2} & 0\\ {}* & * & * & * & * & -\bar{q}I \end{array}\right]<0 \end{array}$$

*where*

$$\begin{array}{cc} \Xi_{i}^{1}= & -P_{i}+\bar{q}\varepsilon C_{i}^{T}C_{i}+I,\\ \Xi_{i}^{2}= & \bar{q}\varepsilon D_{i}^{T}D_{i}-\gamma^{2}I,\\ \Psi_{ij}= & -U_{i}-U_{i}^{T}+P_{j},\forall j\in £,\\ \Phi_{i}^{1}= & \left[\begin{array}{cc} U_{i}^{1}{\bar{A}}_{i}-K_{i}{\bar{C}}_{i} & 0\\ 0 & U_{i}^{2}{\bar{A}}_{i} \end{array}\right],\\ \Phi_{i}^{2}= & \left[\begin{array}{ccc} U_{i}^{1}{\bar{E}}_{i} & -K_{i}F_{i} & 0\\ U_{i}^{2}{\bar{E}}_{i} & 0 & U_{i}^{2}{\bar{B}}_{i} \end{array}\right],\\ \Phi_{i}^{3}= & \left[\begin{array}{c} K_{i}\\ 0 \end{array}\right]. \end{array}$$

*Correspondingly, the observer gain can be calculated by $L_{i}={\left(U_{i}^{1}\right)}^{-1}K_{i},i\in £$.*

**Proof.**  In accordance with the Schur complement property, $\Pi_{i}<0$ equals to

(18) $$\begin{array}{cc} \; & \left[\begin{array}{cccccc} -P_{1} & 0 & \cdots & \sqrt{\mu_{i1}}P_{1}A_{i} & \sqrt{\mu_{i1}}P_{1}B_{i} & \sqrt{\mu_{i1}}P_{1}E_{i}\\ {}* & \vdots & \vdots & \vdots & \vdots & \vdots \\ {}* & * & -P_{N} & \sqrt{\mu_{iN}}P_{N}A_{i} & \sqrt{\mu_{iN}}P_{N}B_{i} & \sqrt{\mu_{iN}}P_{N}E_{i}\\ {}* & * & * & \Xi_{i}^{1} & \bar{q}\varepsilon C_{i}^{T}D_{i} & 0\\ {}* & * & * & * & \Xi_{i}^{2} & 0\\ {}* & * & * & * & * & -\bar{q}I \end{array}\right]<0. \end{array}$$

Performinga congruent transformation on expression (18) by $diag\{P_{1}^{-1},\cdots ,P_{N}^{-1},I\}$ and then performing the same transformation on the resulting form by $diag\{U_{i},\cdots ,U_{i},I\}$, we obtain

(19) $$\begin{array}{cc} \; & \left[\begin{array}{cccccc} {\bar{\Psi }}_{i1} & 0 & \cdots & \sqrt{\mu_{i1}}U_{i}A_{i} & \sqrt{\mu_{i1}}U_{i}B_{i} & \sqrt{\mu_{i1}}U_{i}E_{i}\\ {}* & \vdots & \vdots & \vdots & \vdots & \vdots \\ {}* & * & {\bar{\Psi }}_{iN} & \sqrt{\mu_{iN}}U_{i}A_{i} & \sqrt{\mu_{iN}}U_{i}B_{i} & \sqrt{\mu_{iN}}U_{i}E_{i}\\ {}* & * & * & \Xi_{i}^{1} & \bar{q}\varepsilon C_{i}^{T}D_{i} & 0\\ {}* & * & * & * & \Xi_{i}^{2} & 0\\ {}* & * & * & * & * & -\bar{q}I \end{array}\right]<0, \end{array}$$

where ${\bar{\Psi }}_{ij}=-U_{i}P_{j}^{-1}U_{i}^{T},j\in £$. It is obvious from Lemma 1 that the expression (19) is guaranteed if condition (17) holds. Finally, the conclusion is drawn on the basis of the result in Theorem 1.    □

**Remark** **2.** *To acquire the optimal observer that achieves $H_{\infty }$ performance, we can resort to the subsequent optimization procedure. We aim to minimize the scalar γ, and this minimization is subject to the constraints specified in Equation (17). In essence, this formulation seeks the solution of the following optimization problem*

$$\begin{array}{c} \left\{\begin{array}{c} \min\;\gamma \\ s.t.\;\mathrm{constraints}\;\mathrm{in}\;(17). \end{array}\right. \end{array}$$

### 3.2. Zonotopic Set-Valued Estimation

In this subsection, we are dedicated to developing a zonotopic set-valued estimation approach for Markov jump system via leveraging the acquired $H_{\infty }$ observer. This takes into account uncertainties arising from disturbances, noises, and deviations induced by triggering mechanism.

Considering the dynamics of estimation error system (10), we assume that the disturbances and noises are unknown but bounded, i.e., $|w_{k}|\leq \bar{w},\left|v_{k}\right|\leq \bar{v}$, where vectors $\bar{w}$ and $\bar{v}$ are known. In this case, the uncertainty $d_{k}$ is contained by the following zonotope:

(20) $$\begin{array}{c} d_{k}\in Z_{d}=\langle 0,H_{d}\rangle , \end{array}$$

in which

$$\begin{array}{cc} H_{d}= & \left[\begin{array}{cc} H_{w} & 0\\ 0 & H_{v} \end{array}\right],\\ H_{w}= & diag\left\{\bar{w}\right\},H_{v}=diag\left\{\bar{v}\right\}. \end{array}$$

From the triggering mechanism in (7), it can be derived that

$$\begin{array}{cc} |\delta_{k}|\leq & \bar{\varepsilon }\left|y_{k}\right|\\ = & \bar{\varepsilon }\left|y_{k_{s}}+\delta_{k}\right|\\ \leq & \bar{\varepsilon }|y_{k_{s}}|+\bar{\varepsilon }\left|\delta_{k}\right|, \end{array}$$

thus implying

$$\begin{array}{c} |\delta_{k}|\leq \frac{\bar{\varepsilon }}{1-\bar{\varepsilon }}\left|y_{k_{s}}\right|, \end{array}$$

with $\bar{\varepsilon }=\sqrt{\varepsilon }$. By defining $H_{\delta }=\frac{\bar{\varepsilon }}{1-\bar{\varepsilon }}\cdot diag\{|y_{1}\left(k_{s}\right)\left|,\cdots ,\right|y_{n_{y}}\left(k_{s}\right)\left|\right\}$, we obtain

(21) $$\begin{array}{c} \delta_{k}\in \langle 0,H_{\delta }\rangle . \end{array}$$

Next, the zonotopic state estimation for system (6) is proposed in the following theorem.

**Theorem** **3.** *For σ(k)=i∈£, if ${\bar{x}}_{k}\in \langle {\hat{x}}_{k},H_{k}\rangle$, then*

(22) $$\begin{array}{c} {\bar{x}}_{k+1}\in Z_{x}\left(k+1\right)=\langle {\hat{x}}_{k+1},{\bar{H}}_{k+1}\rangle , \end{array}$$

*where ${\bar{H}}_{k+1}$ is the reduced generator matrix of $H_{k+1}$ and*

(23) $$\begin{array}{c} H_{k+1}=\left[\begin{array}{ccc} A_{e,i}H_{k} & D_{i}H_{d} & L_{i}H_{\delta } \end{array}\right]. \end{array}$$

**Proof.**  Due to $e_{k}={\bar{x}}_{k}-{\hat{x}}_{k}$ and ${\bar{x}}_{k}\in \langle {\hat{x}}_{k},H_{k}\rangle$, we know that $e_{k}\in \langle 0,H_{k}\rangle$. Drawing on the system dynamics described in (10) and making use of the properties in Lemma 2, we arrive at

$$\begin{array}{cc} e_{k+1}\in & Z_{e}\left(k+1\right)\\ = & A_{e,i}⊙\langle 0,H_{k}\rangle ⊕D_{i}⊙\langle 0,H_{d}\rangle ⊕L_{i}⊙\langle 0,H_{\delta }\rangle \\ = & \langle 0,H_{k+1}\rangle . \end{array}$$

Using the reduction operator approach in [32], it holds that $e_{k+1}\in \langle 0,{\bar{H}}_{k+1}\rangle$. Then, together with ${\bar{x}}_{k+1}={\hat{x}}_{k+1}+e_{k+1}$, it is easy to get the inclusion relationship in (22), thereby completing the proof.    □

**Remark** **3.** *Given the order of matrix $H_{k}$, for every $k\in N_{+}$, it rises as an iterative estimation process goes on. From a practical application perspective, the size of the intersection zonotope needs to be restricted. Hence, we introduce the reduction generator matrix ${\bar{H}}_{k}$, which is used to limit the upper bound of the matrix columns and meanwhile maintain the following property*

$$\begin{array}{c} \langle {\hat{x}}_{k},H_{k}\rangle \subseteq \langle {\hat{x}}_{k},{\bar{H}}_{k}\rangle . \end{array}$$

*The specific calculation procedure for the reduced generator matrix is presented in* ***Algorithm 1*** *. Then for $s=1,\cdots ,n_{x}$, the upper bound and lower bound can be calculated as follows:*

$$\begin{array}{cc} x_{s,k}^{upper}= & {\hat{x}}_{s,k}+\underset{k=1}{\sum^{r}}\left|{\bar{H}}_{s,k}\right|,\\ x_{s,k}^{lower}= & {\hat{x}}_{s,k}-\underset{k=1}{\sum^{r}}\left|{\bar{H}}_{s,k}\right|, \end{array}$$

*where r stands for the ideal order in the reduction operator. It is worth mentioning that a larger order r yields a greater computational load but a smaller decline in accuracy. Therefore, a suitable order r needs to be carefully selected to achieve a balance between computational cost and estimation performance in practical applications.*

**Algorithm 1:** Order reduction procedure
**Input:** generator matrix $H_{k}\in R^{n\times t}$ and ideal order *r*

**Process:**

If t≤r, set ${\bar{H}}_{k}=H_{k}$ else if n≤r<t

**(i)** Sort columns of $H_{k}$ in descending order by Euclidean norm, denote the sorted matrix as ${\hat{H}}_{k}$**(ii)** Extract the first *r* columns of ${\hat{H}}_{k}$ to form $H_{k}^{1}$**(iii)** Construct diagonal matrix $H_{k}^{2}$ where $H_{k}^{2}\left(i,i\right)=\sum_{q=r-n+1}^{t}\left|{\hat{H}}_{i,q}\right|$ for i=1,2,⋯,n**(iv)** Define ${\bar{H}}_{k}=\left[H_{k}^{1},H_{k}^{2}\right]$**Output:** reduced generator matrix ${\bar{H}}_{k}$



## 4. Numerical Results

Consider the boost converter system in Section 2, in which the switching is governed by a Markov process between two modes. Let R=1Ω,L=1H,C=1F and redefine the state variable $x\left(t\right)={\left[V_{c},i_{L},1\right]}^{T}$. The system is supposed to be stabilized by certain control rules. With the same sampling and discretization techniques as in [25,33], the commonly used system parameters are obtained as below:

$$\begin{array}{cc} A_{1}= & \left[\begin{array}{ccc} 0.94 & 0.10 & 0.06\\ -0.30 & 0.95 & -0.30\\ -0.25 & -0.06 & 0.63 \end{array}\right],\\ A_{2}= & \left[\begin{array}{ccc} 0.93 & 0.08 & 0.07\\ -0.14 & 0.66 & -0.20\\ -0.16 & -0.4 & 0.66 \end{array}\right], \end{array}$$

which also correspond to the matrices $A_{0,1},A_{0,2}$ in model (4). Additionally, other parameters of the model are taken to be

$$\begin{array}{cc} A_{1,1}= & \left[\begin{array}{ccc} -0.03 & 0.01 & 0.02\\ 0.01 & -0.07 & 0.02\\ 0.03 & 0.05 & -0.3 \end{array}\right],B_{1}=\left[\begin{array}{c} -0.3\\ 0.4\\ 0.6 \end{array}\right],\\ A_{1,2}= & \left[\begin{array}{ccc} -0.01 & 0.02 & 0.04\\ 0.05 & -0.05 & 0.04\\ 0.06 & 0.03 & -0.5 \end{array}\right],B_{2}=\left[\begin{array}{c} 0.2\\ 0.6\\ -0.4 \end{array}\right],\\ E_{1}= & \left[\begin{array}{ccc} 0.2 & 0.1 & -0.1 \end{array}\right],E_{2}=\left[\begin{array}{ccc} -0.2 & 0.5 & 0.3 \end{array}\right],\\ C_{1}= & \left[\begin{array}{ccc} -0.1 & 0.2 & 0.3 \end{array}\right],C_{2}=\left[\begin{array}{ccc} -0.2 & -0.2 & 0.3 \end{array}\right],\\ F_{1}= & F_{2}=0.1. \end{array}$$

Setting the parameters τ=0.6,ε=0.9,θ=5,γ=1.5 and $\Gamma =\left[\begin{array}{cc} 0.5 & 0.5\\ 0.7 & 0.3 \end{array}\right]$, we solve the inequalities in Theorem 2 to obtain the optimal $H_{\infty }$ observer gain matrices as follows:

$$\begin{array}{cc} L_{1}= & {\left[\begin{array}{cccccc} -0.0033 & 0.0055 & 0.0028 & -0.0044 & 0.0061 & 0.0045 \end{array}\right]}^{T},\\ L_{2}= & {\left[\begin{array}{cccccc} -0.0067 & -0.0022 & 0.0034 & -0.0071 & -0.0036 & 0.0041 \end{array}\right]}^{T}. \end{array}$$

**Figure 3:**
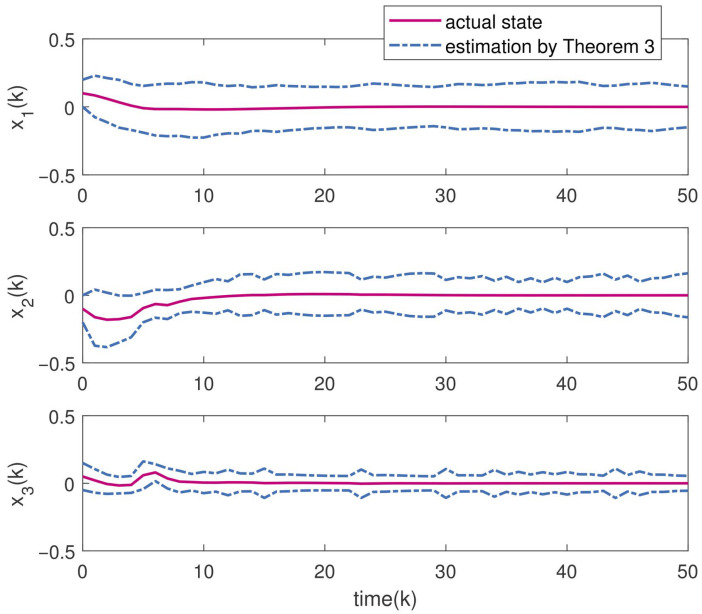
Zonotopic state estimation results by Theorem 3.

**Figure 4:**
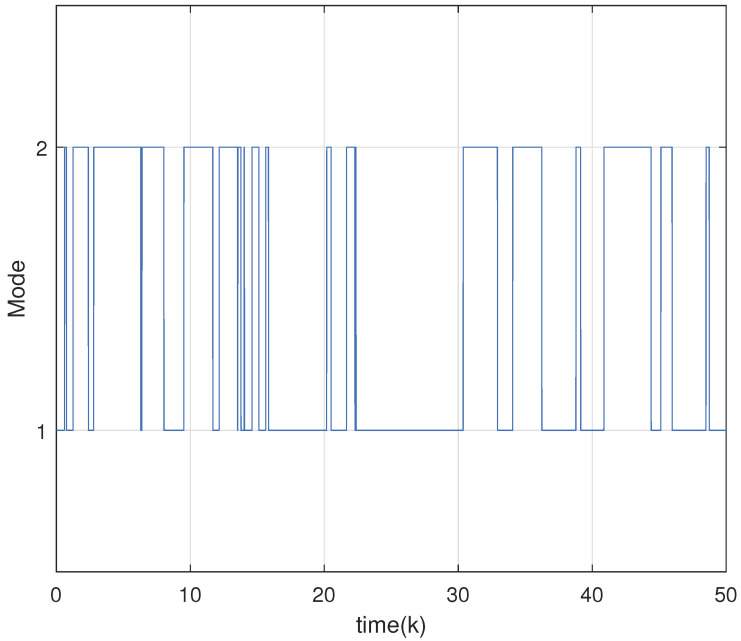
Mode evolution of boost converter system modeled by Markov jump process.

**Figure 5:**
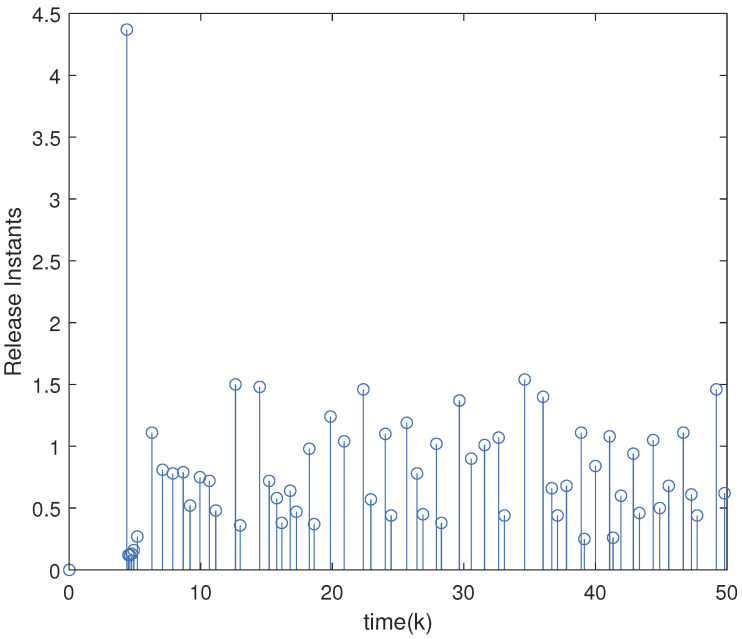
Release instants and intervals by adaptive event trigger (7).

The initial value of the underlying system is $x_{0}={\left[0.1,-0.1,0.05\right]}^{T}$. The total duration of numerical simulation is chosen as 50 with discrete time step 0.1. The uncertainties including external disturbances and noises are bounded within a zonotope $Z_{d}=\langle 0,H_{d}\rangle$ with $H_{d}=diag\left\{0.1,0.1\right\}$. By applying Theorem 3 with the designed optimal $H_{\infty }$ observer gains, the zonotopic state estimation is performed and displayed in Figure 3. The red solid curve denotes the actual system state, and the blue dashed curve represents the estimated boundary. It is clear from the figure that the actual state trajectories are bounded within the zonotopic bounding, which implies the effectiveness of the developed zonotopic estimation method. Moreover, Table 2 displays the interval widths at the final simulation instant (k = 50) by Theorem 3 and interval observer method in [34]. From Table 2, one can see that a tighter bounding can be obtained by Theorem 3 compared with that in [34], which illustrates the superiority of our proposed method. The Markov jump process, which describes the mode evolution of the boost converter system, is illustrated in Figure 4. In addition, the adaptive event-triggered mechanism (7) is implemented to govern data transmission during the estimation process. Figure 5 draws the adaptive triggering intervals and instants, which show that only 62 data measurements are triggered for updating. The occupancy rate of network bandwidth is reduced by over 87.6%, which demonstrates that the adaptive event-triggered mechanism effectively alleviates the communication load.

## 5. Conclusions

This paper focuses on studying the state estimation for boost converter systems via a zonotopic set-membership approach. By modeling the boost converter as a Markov jump system with time delays, disturbances, and noises, and introducing an adaptive event-triggered mechanism for data transmission, a zonotopic set-membership estimation method is proposed. This method includes designing an observer for the augmented system to guarantee $H_{\infty }$ performance and establishing an algorithm to construct zonotopes enclosing all system states. Numerical experiments validate that the proposed approach effectively addresses the challenges posed by the nonlinear dynamics and uncertainties/disturbances of boost converters, providing a robust solution for state estimation in such systems and having potential applications in modern power electronics scenarios like renewable energy integration and electric vehicle charging.

## Figures and Tables

**Table 1:** Notations throughout the paper.

Symbols	Meanings
∥X∥	Euclidean norm of matrix *X*
$X^{T}$	Transpose of matrix *X*
X>0 (<0)	Matrix *X* is positive (negative) definite
*	Wntry induced by symmetric matrix
⊕	Minkowski sum
⊙	Linear image operator
E{·}	Mathematical expectation operator
diag{·}	Block diagonal matrix
$N_{+}$	Positive integer set

**Table 2:** Interval estimation widths by different methods (k = 50).

Methods	Widths
$|x_{k}^{upper}-x_{k}^{lower}|$ by Theorem 3	${\left[\begin{array}{ccc} 0.3431 & 0.1938 & 0.1674 \end{array}\right]}^{T}$
$|x_{k}^{upper}-x_{k}^{lower}|$ by [34]	${\left[\begin{array}{ccc} 0.3819 & 0.3284 & 0.2955 \end{array}\right]}^{T}$

## Data Availability

Included in the article are the original contributions of this research; additional inquiries could be directed to the corresponding authors.
